# Graft failure and revision rate after ACL repair with dynamic intraligamentary stabilization. One-year results of a prospective case series of 155 patients

**DOI:** 10.1186/s40634-023-00614-y

**Published:** 2023-05-05

**Authors:** Ajmal Farid, Sophie A. Gommers, Inger N. Sierevelt, Floor van Eijk, Paulien M. van Kampen, Daniël Haverkamp, Dutch A. C. L. repair study group, Niels Baas, Maaike v/d Borne, Hans Frejlach, Peter Joosten, Tom Hogervorst, Daniël Hoornenborg, Gino Kerkhoffs, Arno van Lieshout, Bart Muller, Marina van Rhee, Harm van der Vis

**Affiliations:** 1 Department of Orthopaedic Surgery, Xpert Clinics Orthopedie, Amsterdam, The Netherlands; 2 Department of Orthopaedic Surgery, Bergman Clinics, Rijswijk, The Netherlands; 3 Spaarne Gasthuis Academy, Orthopedic Department, Hoofddorp, The Netherlands

**Keywords:** ACL repair, Dynamic intraligamentary stabilization, DIS, ACL rupture, Knee, Instability, ACL reconstruction, Ligamys, Anterior cruciate ligament

## Abstract

**Purpose:**

The aim of this study was to assess graft failure, revision rate, and functional outcomes after treatment of acute ACL rupture with dynamic intraligamentary stabilization (DIS) Ligamys device one year post surgery. Additionally, differences in functional outcome between patients with and without anteroposterior laxity were assessed. It was hypothesized that the failure rate of DIS was non-inferior to that of previously reported ACL reconstruction (10%).

**Methods:**

In this prospectively designed multicenter study, including patients with an acute ACL rupture, DIS was performed within 21 days after rupture. Primary outcome was failure of the graft at 1 year post surgery, defined as 1) re-rupture of the graft, 2) revision of DIS, or 3) a > 3 mm side-to-side difference in anterior tibial translation compared to the non-operated knee (∆ATT), measured by the KT1000 device. Additional analysis was performed using a 5 mm threshold. The subjective International Knee Documentation Committee Score (IKDC) and Numerical Rating Scales (NRS) for pain and confidence were used to evaluate functional outcome.

**Results:**

A total of 155 patients were included with a mean age at surgery of 27.8 years (SD 9.4). The mean interval from rupture to DIS was 16.4 days (SD 5.2). At a median follow-up of 13 months (IQR 12–18) the failure rate of the graft was 30.2% (95%CI:22.0–39.4); 11 patients (7%) required secondary reconstructive surgery and of the 105 patients who attended ATT measurement, 24 patients (23%) had an ∆ATT > 3 mm. Secondary analysis, based on a 5 mm threshold, revealed a failure rate of 22.4% (95%CI: 15.2; 31.1). A total of 39 patients (25%) reported at least one complication, comprising mainly arthrofibrosis, traumatic re-rupture and pain. In these patients, removal of the monoblock was performed in 21 cases (13.5%). At follow-up no significant differences in functional outcomes between patients with ∆ATT > 3 mm and stable ATT were observed.

**Conclusion:**

This prospective multicenter study found a high failure rate at one year follow-up of 30% (7% revision surgery and 23% > 3 mm side-to-side difference in anterior tibial translation) in patients treated by primary repair of the ACL with DIS, and did therefore not demonstrate non-inferiority to ACL reconstruction. For patients who did not require secondary reconstructive surgery, this study found good functional outcomes, also in case of persistent anteroposterior knee laxity (∆ATT > 3 mm).

**Level of evidence:**

Level IV.

## Introduction

ACL reconstruction has become a widely accepted treatment option for instability after ACL ruptures and its surgical technique has evolved rapidly in the last decades [1]. Although results of ACL reconstruction are generally good in terms of regaining stability of the knee joint with failure rates between 5 and 15% [2, 3], there is evidence that it does not prevent radiological osteoarthritis (OA) at long term [4, 5]. The occurrence of OA at long term can be attributed to trauma or (persistent) disturbed native knee kinematics after ACL reconstruction [4]. The latter which is associated with (over) tensioning of the graft and other factors such as the removal of the ACL remnants along with its nerve endings and thus proprioception [6–8]. As the ACL has some self-healing potential [9, 10], remnant preserving surgery, such as ACL repair, could therefore be considered as a plausible option to preserve proprioception and possibly even decrease the risk of OA at long term. As ACL repair has to be performed in the early phase in selected patients, it could have additional beneficial effect on the knee joint, as early ACL surgery is associated with lower risk of meniscal damage and cartilage injury [11].

A relatively new surgical technique applies dynamic augmentation and stabilization of primary ACL repair [12, 13]. This dynamic intraligamentary stabilization (DIS) allows full range of motion and full weight bearing directly after surgery while protecting the sutured ACL [9, 14–16]. It addresses the advantages of ACL repair to remain proprioception of the knee, and is an early surgical intervention (within 21 days after the trauma) allowing optimal early treatment of menisci and/or cartilage [9]. Initial studies showed potential efficacy and safety [12, 13], and multiple single center studies have shown varying results [13, 17–19]. Therefore, this prospective multicenter study in the Netherlands monitored the introduction of DIS into Dutch practice.

The primary aim of this study was therefore to assess graft failure (re-rupture, revision of DIS, or > 3 mm side-to-side difference in anterior tibial translation (∆ATT)), reoperation, and functional outcomes one year post treatment of acute ACL rupture with the DIS Ligamys device. Additionally, differences in functional outcomes between patients with and without anteroposterior laxity (∆ATT > 3 mm) were evaluated. It was hypothesized that the failure rate of DIS was non-inferior to that of previously reported ACL reconstruction (ACLR) of 10% [3].

## Materials and methods

### Study design

The study was designed as a prospective case series assessing anteroposterior (AP) knee laxity and functional outcomes after treatment of acute ACL rupture with ACL suture repair with DIS. The study was conducted in 5 different centers in the Netherlands and was approved by the medical ethical committee (P1503/2014–903) and central committee on research involving human subjects (NL51958.048.14). The study was registered before initiation in the Dutch trial register (NTR7486).

### Patient population

Patients were included in case of an acute primary rupture of the ACL confirmed by MRI, age between 18 and 50 years, surgical intervention planned within 21 days, and a BMI < 35. Patients were excluded in case of osteoarthritis on conventional x-ray (Kellgren-Lawrence grade ≥ 2), traumatic cartilage lesion requiring cartilage repair procedure or degenerative cartilage lesions (Outerbridge grade > 2 and defect size > 1cm2), combined ligament injury, pregnancy, rheumatoid arthritis, instability of the contralateral leg, or unwillingness to follow the rehabilitation program.

### Procedure

After confirmed eligibility and signed informed consent, patients were invited to complete questionnaires electronically at baseline (preoperative) and at 3, 6 and 12 months follow-up. At one year follow-up, patients were invited to the outpatient clinic for AP laxity measurement of the knee joint (time frame 8–18 months). Additionally, patients' files were used to collect information regarding complications and re-operations.

### Surgical technique

All DIS procedures were performed arthroscopically across five participating centers by experienced ACL surgeons using a previously described surgical technique [20]. The ruptured ACL was sutured and stabilized by use of a polyethylene braided cord that was anchored on the antero-medial aspect of the tibia by a Monoblock, a spring-screw implant (Ligamys, Mathys Ltd., Bettlach, Switzerland). The cord was pre-loaded with 60–80 N of force to prevent anterior subluxation of the tibia [21]. In case of concomitant meniscus injuries, partial debridement or repair was performed where necessary.

Postoperative rehabilitation was performed according to physiotherapy rehabilitation guidelines codependent on concomitant meniscal lesions. In case of isolated ACL rupture, full weight bearing was allowed using a brace in the first week. Strength training started in the third week and running and sport specific training exercises were allowed at 10 weeks after surgery.

### Outcome measures

The primary outcome measure of this study was failure of the graft which was defined as 1) re-rupture of the graft, confirmed by Magnetic Resonance Imaging (MRI), 2) revision of DIS (conversion to ACL reconstruction), or 3) a > 3 mm side-to-side difference in anterior tibial translation (ATT) compared to the non-operated knee [3]. The ATT was measured by use of the KT1000 arthrometer (MEDmetric Corp., San Diego, CA, USA) at one year post surgery. This non-invasive test is performed at 20° to 30° knee flexion with 133N anterior force. After preconditioning, the injured and non-injured knee were measured three times. The mean side-to-side difference in ATT between the respective knee joints (∆ATT) was defined as outcome with a value of 3 mm as threshold for instability based on IKDC criteria (IKDC grade B, C and D) [22]. Additional failure analysis was performed using a ∆ATT of 5 mm as threshold (IKDC grade C and D). Since in the literature there is discussion on the threshold we chose for the most strict threshold, but also included a more liberal threshold.

Secondary outcomes were complications, re-operation, and patient reported outcome measures (PROMs) such as the subjective International Knee Documentation Committee Score (IKDC) and Numerical Rating Scale (NRS) for pain during rest and sports activity and a (0 [worst] to 10 [best]) NRS for confidence.

### Sample size calculation

Sample size calculation was based on AP knee laxity measurements using the KT1000, with a 3 mm side-to-side difference threshold as cut-off value to define failure [9, 14, 23]. Assuming a 10% failure rate of the graft in case of ACL reconstruction [3], a non-inferiority limit of 20%, and a one-sided significance level of 0.025 (using Clopper-Pearson confidence interval), 142 patients would be required to confirm non-inferiority to ACL reconstruction with a power of 90%.

### Statistical analysis

Statistical analysis was performed by use of SPSS 26.0 (IBM Corp., Armonk, NY, USA). Continuous variables are described as means with SD in case of normal distribution, otherwise medians with IQRs are presented. Categorical data are described as numbers with accompanying proportions. Failure rate was calculated as a proportion with binomial Clopper-Pearson 95% confidence interval (95%CI) to assess non-inferiority [24]. DIS repair was considered non-inferior to ACL reconstruction if the upper boundary of the two-sided 95%CI lay beneath the predefined non-inferiority margin of 20%. Revision rates at 12 and 18 months were assessed by use of Kaplan Meier survival analysis and presented with 95%CIs. Among patients without revision surgery, differences in IKDC scores between DIS failure (∆ATT > 3 mm) and non-failure group (∆ATT ≤ 3 mm) were assessed by use of Students' t-tests at baseline and at primary endpoint of 12 months follow-up. Additional mixed model analyses were performed to assess crude as well as adjusted (for age, gender and BMI) differences between the groups during 12 months follow-up.

Due to skewed distributions and outliers, NRS-scales were analyzed non-parametrically using Mann Whitney U-tests to assess differences between the two groups at both time points. Change during follow-up was tested by use of Friedman tests with imputation according to the Last observation carried forward protocol (LOCF). An additional per protocol sensitivity analysis was performed. A p-value < 0.05 was considered statistically significant.

## Results

### Patient characteristics

One hundred and fifty-five patients were included in the study across five participating centers. Mean age at surgery was 27.8 years (SD 9.4) and the majority (61%) was male. The mean time interval from injury to surgery was 16.4 days (SD 5.2). Both menisci were undamaged in 64 patients (41%) and the ACL tear was mainly located in the proximal third (63%) (Table 1). Of these 155 patients, 39 (25%) were lost for stability measurement and PROMs at follow-up.

**Table 1:** Patient demographics and peroperative details of the study population (*n* = 155)

Gender		
Male	94	(61%)
Female	61	(39%)
Age at inclusion (years)		
Mean (SD)	27.8	(9.4)
BMI (kg/m^2^)		
Mean (SD)	24.3	(3.4)
Medial Meniscus		
Healthy	126	(81%)
Partial resection	6	(4%)
Meniscal Repair	23	(15%)
Lateral Meniscus		
Healthy	92	(59%)
Partial resection	43	(28%)
Meniscal Repair	20	(13%)
Cartilage defect		
None	148	(95%)
Micro# or debridement	7	(5%)
Tear location		
Proximal	97	(63%)
Central ^1^/_3_	39	(25%)
Distal	1	(1%)
Rupture Pattern		
Single strand	39	(25%)
2 bundles	67	(43%)
3 or more strands	26	(17%)
Synovial sheath		
Completely intact	57	(37%)
> 50% intact	48	(31%)
< 50% intact	12	(8%)

### Graft failure

At a median follow-up of 13 months (IQR 12–18), revision surgery was performed in 11 patients (7%) due to a traumatic re-rupture, confirmed by MRI or arthroscopy (*n* = 10) or clinical failure (instability) of the graft (*n* = 1). One hundred and five patients (68%) had attended their ATT measurement in whom persistent AP laxity (∆ATT > 3 mm) was found in 24 patients (23%). As ATT measurements of 39 patients were not available, this resulted in 35 failures in 116 patients, implying a graft failure rate of 30.2% (95%CI: 22.0- 39.4). Best-case scenario analysis, assuming 35 failures in all 155 patients, resulted in a failure rate of 22.6% (95%CI: 16.3; 30.0). Patients with a failed graft were significantly younger than patients with an intact graft at follow-up (mean age 24.1 (SD 8.9) vs 29.1 (SD 10.3), *p* = 0.02).

Additional analysis based on the same failure definition but with a 5 mm threshold for failure revealed 15 patients (15%) with persistent AP laxity (∆ATT > 5 mm), resulting in 26 failures implying a graft failure rate of 22.4% (95%CI: 15.2; 31.1). According to the best-case scenario this failure rate would be 16.8% (95%CI:11.3; 23.6). In all cases non-inferiority to ACL reconstruction (10% failure rate) was not confirmed.

### Patient reported outcome measures

**Fig. 1:**
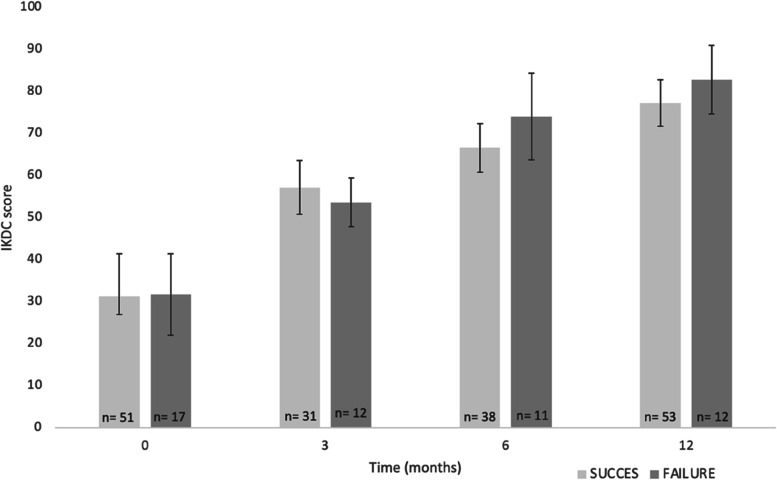
IKDC score during follow-up. Data are presented as means (with 95% CI). Failure is defined as ∆ATT > 3 mm at follow-up

A total of 115 (74%) patients completed PROMs during follow-up, of which 87 (76%) patients had filled out baseline PROMs (Table 2). An overall significant improvement during follow-up was observed for the IKDC (Fig. 1) as well as the NRS scores (*p* < 0.01 for all tests). Among the patients with KT-1000 measurements of whom PROMs were available (*n* = 86, 19 failures, 67 successes), crude and adjusted differences in IKDC score between patients who were considered failures and those with successful repairs during follow-up were 1.7 (95%CI -5.7; 9.3) and 1.3 (95%CI -6.6; 9.2), which were not statistically significant (*p* = 0.65 and *p* = 0.75, respectively). At baseline and 12 months follow-up, no significant differences in all PROM scores between the two groups were observed (Table 2).

**Table 2:** PROMs at baseline and 12 months follow-up of patients without revision surgery

	Total population		Success^a^ ∆ATT ≤ 3 mm		Failure^a^ ∆ATT > 3 mm		*P*-value
	N		N		N		
**IKDC**, mean (SD)							
*Baseline*	87	31 (21; 45)	51	31 (16)	17	31 (18)	0.98
*12 months*	80	87 (74; 94)	53	77 (20)	12	82 (13)	0.36
**NRS** rest, median (IQR)							
*Baseline*	86	2 (0; 4)	50	2 (1; 5)	17	2 (1; 4)	0.73
*12 months*	80	0 (0; 0)	53	0 (0; 1)	12	0 (0; 0)	0.09
**NRS** sport, median (IQR)							
*Baseline*	86	9 (6; 10)	51	9 (7; 10)	16	10 (6; 10)	0.61
*12 months*	80	1 (0; 4)	53	1 (0; 4)	12	1.5 (0; 4)	0.70
**NRS** confidence, median (IQR)							
*Baseline*	78	2 (1; 4)	46	2 (1; 4)	16	2 (0; 4)	0.38
*12 months*	80	8 (5; 10)	53	8 (4; 10)	12	8 (4; 10)	0.68

*IKDC* International Knee Documentation Committee Score, *NRS* numerical rating scale

^a^Failure and success defined at follow-up

### Complications and re-interventions

As documented in patients' files, 39 out of 155 patients (25%) presented with at least one complication up to 18 months follow-up. Complications comprised mainly arthrofibrosis, traumatic re-rupture and persistent pain at the site of the monoblock. Almost all complications required re-intervention (Table 3).

**Table 3:** Complications and re-interventions (*n* = 155)

Complications		
Arthrofibrosis/cyclops	11	(7.1%)
Aseptic loosening of the monoblock^a^	1	(0.6%)
local infection at the level of the monoblock	2	(1.3%)
Septic arthritis of the knee	1	(0.6%)
Meniscal tear	3	(1.9%)
Thrombosis	2	(1.3%)
Persisting pain (due to monoblock)	12	(7.7%)
Re-rupture (traumatic)	10	(6.5%)
Severe instability	1	(0.6%)
Overall complication rate (patients)	39	(25.2%)
Re-interventions		
Revision ACL reconstruction	11	(7.1%)
Removal monoblock	21	(13.5%)
Repeat arthroscopy	12	(7.7%)
MUA	2	(1.3%)
Overall re-interventions (patients)	36	(23.2%)

^a^after peroperative tibial fracture

*ACL* anterior cruciate ligament, *MUA* movement under anesthesia

#### Revision surgery

**Fig. 2:**
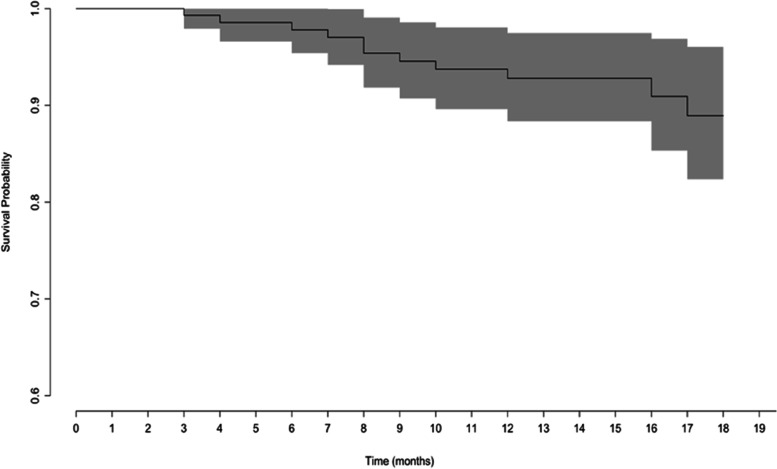
Survival curve for revision surgery for re-rupture or persistent instability

Eleven (7.1%) patients presented with a clinical failure of the ACL repair due to either a traumatic re-rupture (*n* = 10, 6.5%) or severe persistent instability (*n* = 1, 0.6%). All patients needed secondary reconstructive surgery. Based on KM survival analysis, the 12- and 18-months revision rates due to re-rupture or clinical failure were 6.3% (95%CI: 2.2- 10.4) and 10.1% (95%CI: 3.2–17.0), respectively (Fig. 2).

#### Removal of the monoblock

Additional to the 11 revisions (with monoblock removal), the monoblock was removed in 21 (13.5%) patients as a result of local pain or discomfort (*n* = 12, 7.1%), local infection (*n* = 1, 0.6%), septic arthritis (*n* = 1, 0.6%), aseptic loosening after a per-operative tibial fracture at the level of the monoblock (*n* = 1, 0.6%), and during arthroscopy for cyclops syndrome or arthrofibrosis (*n* = 6, 3.9%).

#### Other interventions

Other interventions. Repeat arthroscopy was performed in 12 patients (7.7%) for other reasons, mainly meniscal injuries and cyclops formation (Table 3).

Additionally, movement under anesthesia (MUA) was done in two patients for persistent severe restricted range of motion due to arthrofibrosis. In one of these patients MUA was performed additional to arthroscopic release and removal of the monoblock.

## Discussion

In this Dutch prospective multicenter study, non-inferiority of ACL repair with DIS to ACL reconstruction (ACLR) was not confirmed. Graft failure, as defined by re-rupture, revision of the ACL or persistent objective AP laxity (∆ATT > 3 mm), was observed in 30% of the patients at a median follow-up of 13 months after surgery. Among these patients, revision due to re-rupture or clinical failure, was observed in 7% and persistent AP knee laxity in 23% of the patients. Despite persistent objective AP laxity (∆ATT > 3 mm), good functional outcomes measured by the IKDC and NRS scales were reported. Additional failure analysis, based on the failure definition with a 5 mm threshold, showed that graft failure was present in 22% of the patients.

Although a failure rate of 30% at a median follow-up of 13 months (IQR 12 to 18) is high, the definition of failure should be taken in consideration in the interpretation of the results. In our study strict criteria for failure were applied, comprising a 3 mm threshold for AP laxity of the knee joint in addition to clinical failure and secondary conversion to ACLR. This definition was equal to that of Schmücker et al. who reported failure rates of 9.4% and 11.1% in a retrospective registry study on ACL reconstruction in 475 patients by use of a quadriceps tendon or hamstrings tendon graft, respectively [3].

However, due to considerable variation in graft failure definition and follow-up, comparison of results between studies may be hampered. AP laxity thresholds of 3 mm and 5 mm are both reported in literature [3, 13, 14, 19, 25–27]. Based on the threshold of 5 mm, Lindanger et al. found a failure rate of 9% at 2 years after ACL reconstruction, which is substantially smaller than the 22% found in our study. Köster et al. and Glassbrenner et al. considered a laxity measurement of more than 3 mm side-to-side difference only as clinical failure if the patient also reported subjective knee instability [25, 26]. Alternatively, Kohl et al. reported a positive pivot shift as indicator for failure [13].

Additional to ACL revisions, 23% of the patients showed persistent AP knee laxity in our study (∆ATT > 3 mm), which is slightly higher than the results of Seftl et al. who reported persistent AP laxity in 18% of the patients [19]. However, Kösters et al. reported only 7% persistent laxity at two years [26]. In both studies these patients did not suffer subjective instability and a threshold of 3 mm was used. In an RCT of Glassbrenner et al. ACL repair with DIS was compared to ACLR in 85 patients. They reported high rates of recurrent instability in both the DIS group (35%) and the ACLR group (20%) at 27 and 15 months follow-up, respectively, and concluded that young age and high pre-injury activity level were important risk factors for secondary failure [25]. This was confirmed in a review of Wiggins et al. [28]. Although activity level was not reported in our study, the effect of age was comparable. However, as the mean age of the entire patient population (mean 28 years) was comparable to other studies [3, 19, 25, 26, 28], our high failure rates could not be attributed to younger age.

As knee laxity is more likely to influence knee kinematics than knee stability as subjectively evaluated by the patient, it emphasizes the need for additional objective stability measurements of the knee joint. However, measurement of AP knee laxity is a measure of static stability neglecting the proprioception which is an important condition for the overall functional results of ACL reconstruction [29]. This could explain good functional outcomes despite the presence of persistent laxity of the knee joint in 23% of the patients. The high IKDC scores in this study were in line with other studies using DIS and did not confirm the association between persistent laxity and inferior clinical outcome [13, 19, 26].

Conversion to ACL reconstruction is often used as primary endpoint after ACL repair with DIS at varying follow-up moments [13, 14, 18–20, 26, 30]. The current study found a one-year KM revision rate of 6.3%, which is comparable to Büchler et al. and Kohl et al. who reported revision rates of 6.7% and 8% after DIS at one year follow-up, respectively [13, 20]. Studies with longer follow-up reported higher revision rates, such as 18% at two years up to 23% at 5-year follow-up [14, 19, 26]. A review of Crawford et al. reported a re-rupture rate around 6% after ACL reconstruction at 10 years follow-up [31].

Based on the size of the tibial spring-screw implant (monoblock), discussion was raised about possibilities and success of revision surgery in the case of a failure or re-rupture. In this study, no technical problems with revision surgery were encountered and single stage revision was possible in most cases. This is similar to findings in the literature. In a study by Kösters et al., comparing DIS to ACL reconstruction, two stage revision was necessary for 80% of ACL reconstruction revision cases, while this was never necessary in the DIS group [26]. However, to successfully perform a revision after failed DIS, care should be taken with primary implantation. Glasbrenner et al. showed that, in a porcine knee model, when a bone bridge of about 20 mm is left between the monoblock and tibial joint line knee stability of a single stage revision is comparable to that of a primary ACL reconstruction [32].

Inconclusive policy about removal of the monoblock is represented by the variety of the prevalence of this intervention across studies on DIS. In the present study the monoblock was removed in 13.5% of the patients due to clinical symptoms. Henle et al. reported a removal rate of 24% of whom 10% without symptoms [18], and Senftl et al. presented a 62% removal as they actively offered patients the intervention [19].

Other specific complications in this study were the occurrence of extension deficit and cyclops formation requiring subsequent surgery. These complications are previously reported and authors mention similar rates of occurrence. Kohl et al. reported 10% of cyclops formation limiting full extension in their series [13]. Although this complication is also reported after ACL reconstruction, the prevalence is lower with reported complication rates between 2.4% and 5% [3]. Prevention of this complication needs therefore attention in further studies.

The strength of this study was its prospective design with multiple participating centers which combined objective laxity measurements with subjective PROMs. However, 23% of the study population did not attend their ATT measurements and a considerable number of patients did not complete the questionnaires, which could have caused selection bias. Additionally, the study did not control for surgeon's experience or varying rehabilitation protocols among centers. Results should therefore be interpreted with cause. As the DIS procedure is relatively new, follow-up of the study was short, and long-term effects on the knee joint could not be assessed. Further research should focus on long-term effects of this procedure, and what patient population is suitable for DIS compared to conservative treatment and/or ACL reconstruction.

## Conclusion

This prospective multicenter study found a high failure rate at one year follow-up of 30% (7% revision surgery and 23% > 3 mm side-to-side difference in anterior tibial translation) in patients treated with primary repair of the ACL with DIS, and did therefore not demonstrate non-inferiority to ACL reconstruction. For patients who did not require secondary reconstructive surgery, this study found good functional outcomes, also in case of persistent anteroposterior knee laxity (∆ATT > 3 mm).
